# Supplementary material for: Satb2 acts as a gatekeeper for major developmental transitions during early vertebrate embryogenesis
Source: Nat Commun. 2021 Oct 19;12:6094. doi: 10.1038/s41467-021-26234-7 (PMC8526749; doi:10.1038/s41467-021-26234-7)
Supplement: Supplementary file 1 — Supplementary Information [file 41467_2021_26234_MOESM1_ESM.pdf]

Supplementary Information

Satb2 acts as a gatekeeper for major developmental transitions during early vertebrate embryogenesis

Saurabh J. Pradhan[1], Puli Chandramouli Reddy[1], Michael Smutny[2,3], Ankita Sharma[1], Keisuke Sako[2], Meghana S. Oak[4], Rini Shah[1], Mrinmoy Pal[1], Ojas Deshpande[1,5], Greg Dsilva[1] , Yin Tang[6], Rakesh Mishra[7], Girish Deshpande[8], Antonio J. Giraldez[6], Mahendra Sonawane[5], Carl-Philipp Heisenberg[2]*, and Sanjeev Galande[1,9]*

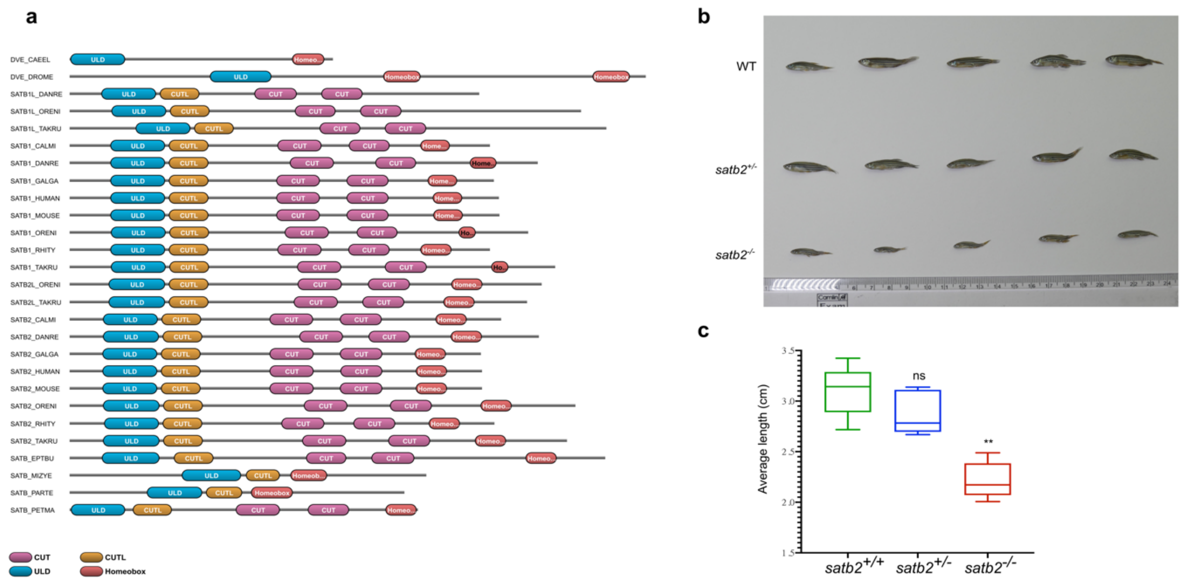

Supplementary Fig. 1. Domain architecture analysis of SATB proteins and characterization of Satb2 mutants. a, Comparative analysis of domain architecture of SATB1 and SATB2 for various species across evolution. ULD domain is represented in blue, CUT like domain in yellow, CUT domain in purple and Homeobox domain in red. Homeobox domain with black text indicates putative or predictive Homeobox. **b,** Digital snapshots of 4 months old zebrafish s*atb2* mutants along with wild-type and heterozygous siblings highlighting defects in body growth. **c,** Box plot indicating the difference in body length between s*atb2* mutants and siblings. ** indicates p-value = 0.0079 as calculated by the student's two-tailed t-test, N=5 individual organisms. Error bar indicates +/- SEM. The whiskers show the minima to the maxima values and the central line indicates the median, *satb2*$^{+/+,}$ 3.143, *satb2*$^{+/-}$ *2.783, satb2*$^{-/-}$ 2.172.

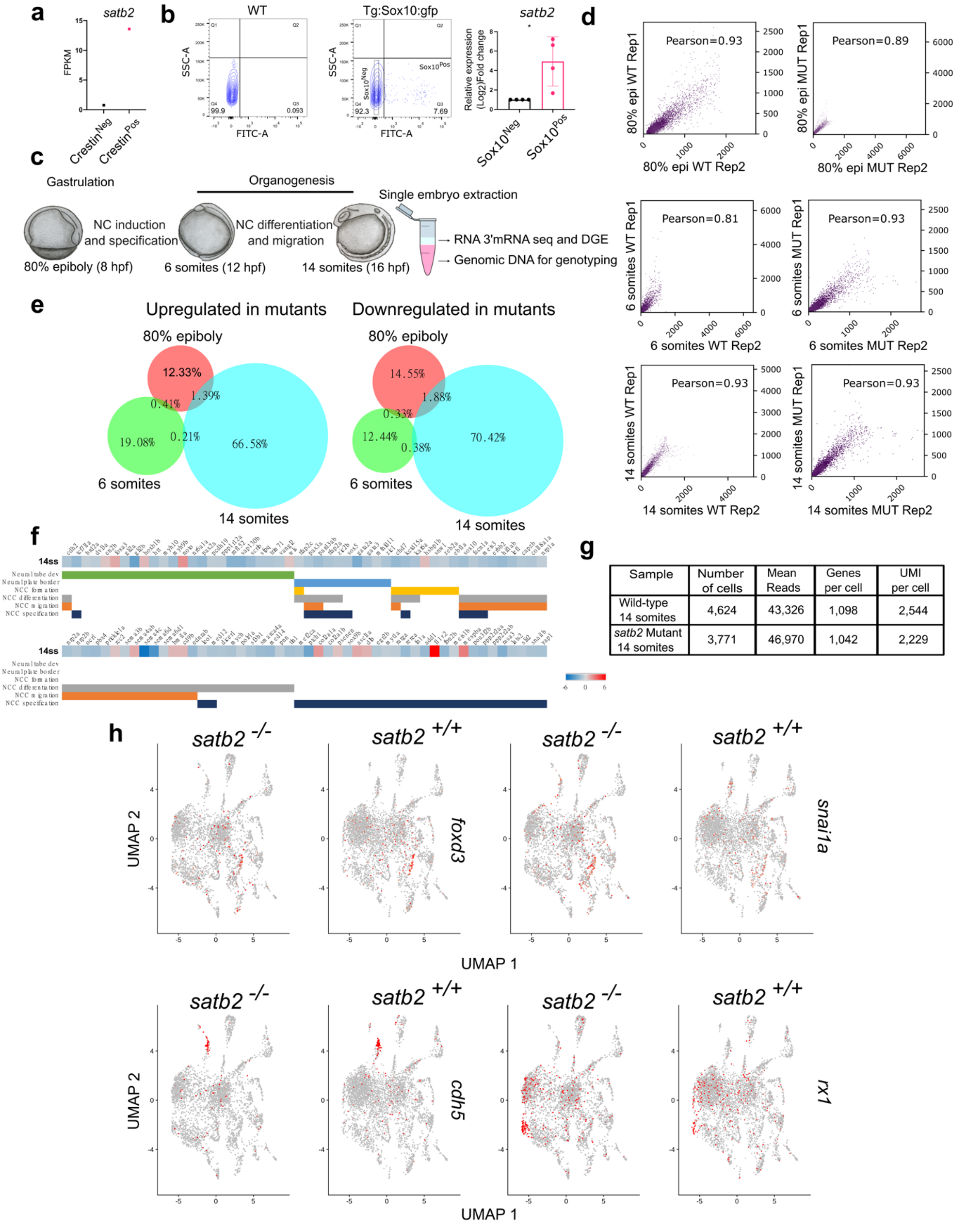

Supplementary Fig. 2. Correlation analysis between replicates of 3' mRNA seq experiments confirming reproducibility and classification of DE genes at 14 somites. a, Dot plot representing FPKM values for *satb2* in Crestin^{Pos} and Crestin^{Neg} cells (GSE75356) b, Contour plots depicting sorting strategy for Sox10^{Neg} and Sox10^{Pos} (7.69%) cells using Tg:Sox10:gfp line by FACS. Dot plot representing $\log_2$ fold change expression for *satb2* in Sox10^{Pos} and Sox10^{Neg} cells as determined using qRT-PCR. 18s rRNA was used as an

endogenous control. * Represents p-value = 0.0208 as calculated by the Student's two-tailed t-test, N=4 independent biological experiments. Error bar indicates +/- SEM. **c,** Experimental strategy for gene expression analysis using 3'mRNA sequencing at 80% epiboly, 6 ss and 14 ss. **d,** Pearson correlation analysis between replicates of transcriptome samples at the corresponding stages suggesting high reproducibility between the replicates. **e,** Venn diagram showing percentage overlap between differentially expressed genes in *satb2*$^{-/-}$ at different stages. **f,** Heatmap of DE genes in 14 ss mutants classified according to their known function during neurogenesis. Genes involved in neural tube development are marked using a green bar, genes involved in neural plate border marked in blue, in NCC formation by yellow, in NCC differentiation by grey, in NCC migration by orange and in NCC specification by dark blue. Color bar indicates the scale of $\log_2$ fold change expression values compared to wild type. **g,** Table summarizing statistics associated with scRNAseq experiment for *satb2*$^{-/-}$ *and satb2*$^{+/+}$ embryos at 14 ss. **h.** UMAP representing expression pattern for key down regulated genes and cell clusters *foxd3*^{Pos} (muscle progenitors and neural crest), *cdh5*^{Pos} (endothelial cell progenitors) and upregulated genes and cell clusters *snai1a*^{Pos} (muscle progenitors), *rx1*^{Pos} (optic progenitor cells).

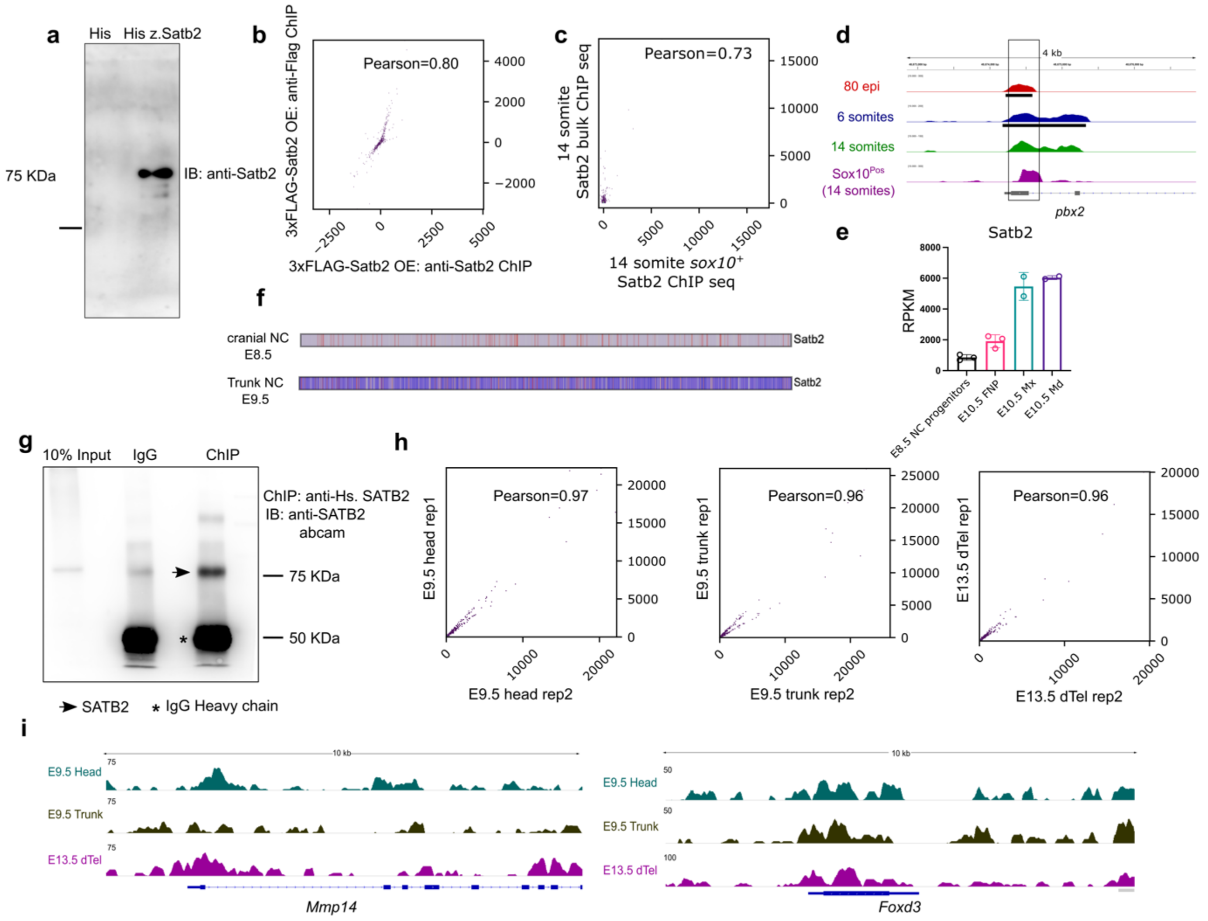

Supplementary Fig. 3. Characterization of anti-SATB2 antibodies and Correlation analysis of zebrafish Satb2 and mouse *SATB2* ChIP seq experiments. a, Immunoblot analysis of recombinant control His tag and His-tagged zebrafish Satb2 protein using lab generated zebrafish specific anti-Satb2 antibody. N=2 biologically independent experiments. **b,** Pearson correlation analysis of ChIP-sequencing datasets obtained using anti-FLAG antibody and anti-SATB2 antibody showing a high level of correlation. **c,** Pearson correlation analysis of ChIP-sequencing datasets obtained using anti-SATB2 antibody at 14 ss embryos and Sox10^{Pos} (neural crest) sorted cells showing a high level of correlation across genomic binding sites identified through 14 somites whole embryo ChIPseq. **d,** IGV snapshots of Satb2 occupancy on genomic loci of zebrafish *pbx2* gene at 80 % epiboly (red), 6 somites (blue), 14 somites (green) and in Sox10^{Pos} (purple) cells. Input subtracted tracks are used for visualization. For Satb2 ChIP seq in Sox10^{Pos} (neural crest) cells, ChIPseq reads are normalized to the signal from Sox10^{Neg} (non-neural crest) cells to depict enrichment at respective gene loci. Solid line boxes highlight enriched genomic regions across all the datasets. **e,** Expression level of Satb2 represented as counts per million in neural crest progenitors at E8.5, N=3, Frontonasal population (FNP), N=3, maxillary (Mx), N=2 and mandibular (Md), N=2 population from publicly available dataset (GSE89434). Error bar represents +/- S.D. **f,** Heatmaps representing expression of Satb2 from scRNAseq data for cranial neural crest cells (E8.5) and Trunk neural crest cells (E9.5) suggesting exclusive expression of Satb2 in cranial neural crest cells (GSE129114). **g,** ChIP western analysis to validate the efficiency of mammalian anti-SATB2 specific antibodies. N=2 biologically independent experiments. **h,** Pearson correlation plots highlighting reproducibility between replicates of mousse ChIP-sequencing samples at corresponding stages and tissues. **i,** IGV snapshots of SATB2 occupancy on genomic loci of mouse *Mmp14* and *Foxd3* genes.

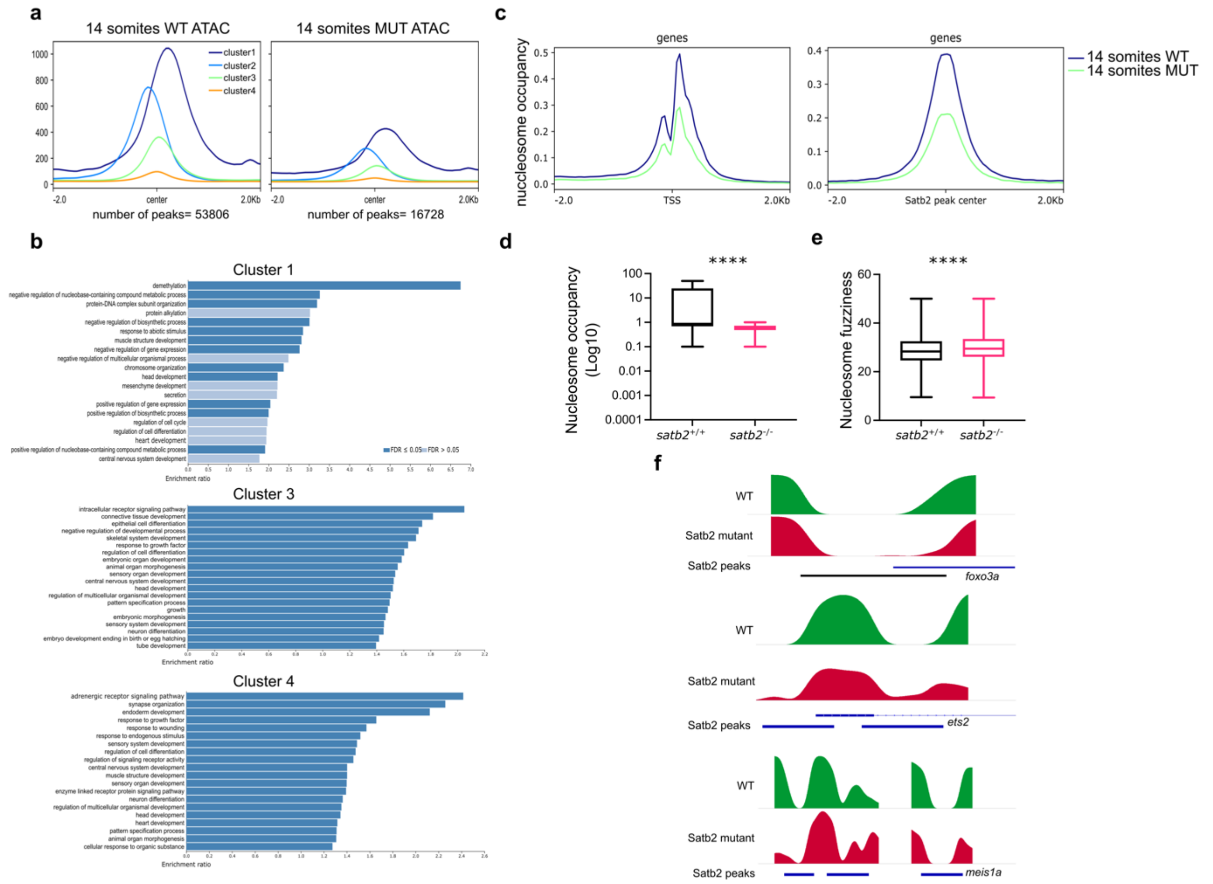

Supplementary Fig. 4. Characterization of chromatin accessibility in s*atb2* mutants compared to wild-type siblings at 14 somites stage. a, K-means clustering of average profiles of chromatin accessibility for wild-type and *satb2*$^{-/-}$ at 14 ss. **b,** Gene ontology analysis for cluster1, cluster3 and cluster4 for K-means clustering of chromatin accessibility. **c,** Mean density maps for average nucleosome occupancy profiles in wild-type and s*atb2* mutants centred around TSS and Satb2 ChIP peak centre respectively. **d,** Dot plot depicting a decrease in average nucleosome occupancy in s*atb2* mutants as compared to wild type. **** p-value 0.0001 as estimated using Student's unpaired t-test. The whiskers show the minima to the maxima values and the central line indicates the median Log10, *satb2*$^{+/+,}$ 0.6038, *satb2*$^{+/-}$ 0.6014, **e,** Dot plot depicting an increase in average nucleosome fuzziness in s*atb2* mutants as compared to wild type. **** p-value 0.0001 as estimated using Student's unpaired t-test. The whiskers show the minima to the maxima values and the central line indicates the median, *satb2*$^{+/+,}$ 28.31, *satb2*$^{+/-}$ 29.49, **f,** IGV snapshots of nucleosome occupancy tracks on the genomic loci of upregulated genes *foxo3a*, *ets2* and *meis1a* upon loss of Satb2.

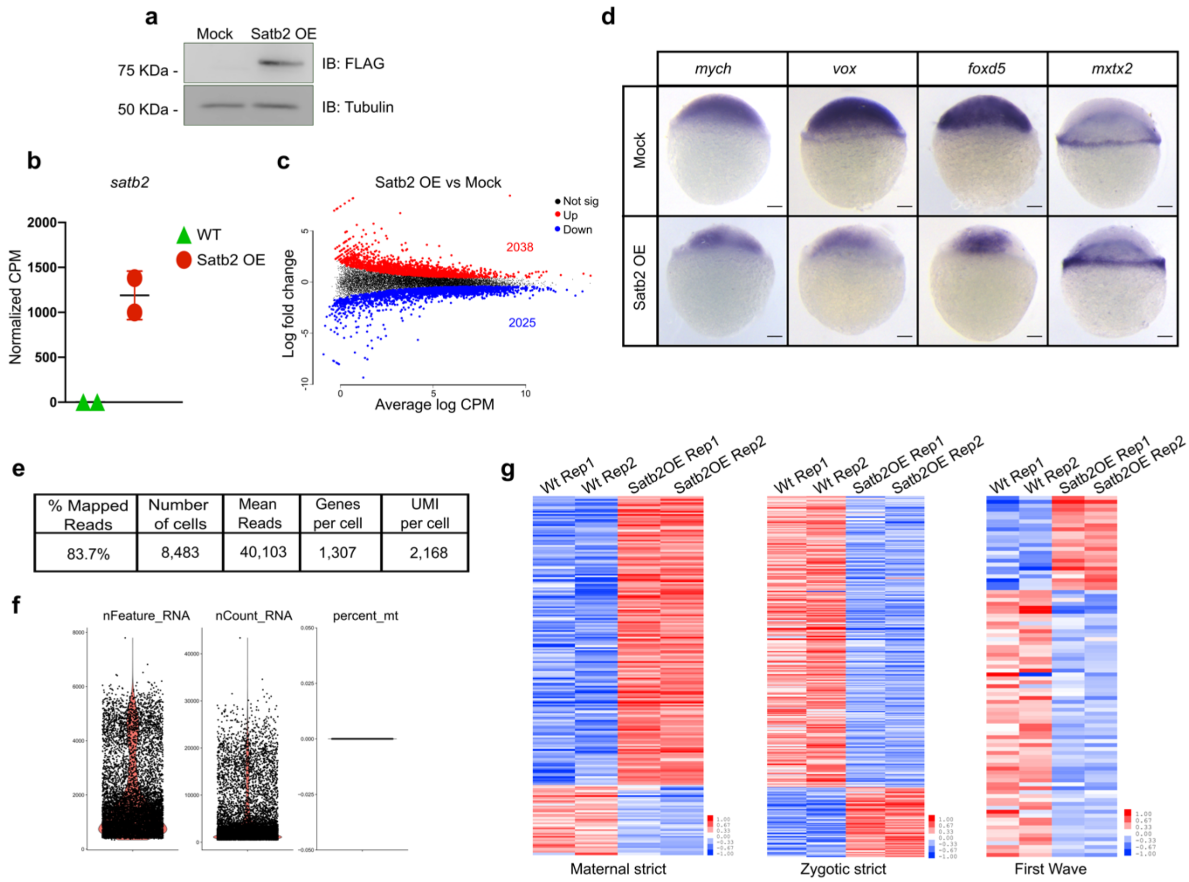

Supplementary Fig. 5. Characterization of the gain of function of maternal Satb2. a, Immunoblot analysis for validating overexpression of 3x-FLAG Satb2 at dome stage using anti-FLAG antibody. gamma-Tubulin was used as a loading control. N=4 biologically independent experiments. Error bar represents +/- S.D. **b,** Dot plot representing and confirming overexpression of Satb2 from RNAseq studies performed at the dome stage (4.5 hpf). Normalized CPM counts were used for the analysis. N=2 biologically independent experiments. +/- SD. **c,** Smear plot analysis for bulk mRNA seq analysis in Mock and 3xFLAG-Satb2 injected embryos at 4.5 hpf. Values in red and blue represent the number of genes differentially upregulated and downregulated respectively. **d,** Whole-mount *in situ* mRNA analysis showing the effect of Satb2 overexpression on expression of *mych*, n=7, N=3, *vox,* n=6, N=3, *foxd5,* n=8, N=3 (downregulated genes) *mxtx2,* n=7, N=3 (upregulated upon overexpression). Scale bar = 100 µm. **e,** Table representing statistics associated with scRNAseq experiment. **f,** Violin plot for visualization of QC metrics of single-cell transcriptome study. **g,** Heatmaps representing differential expression patterns for strictly maternally expressed genes, strictly zygotically expressed genes and first wave zygotic genes.

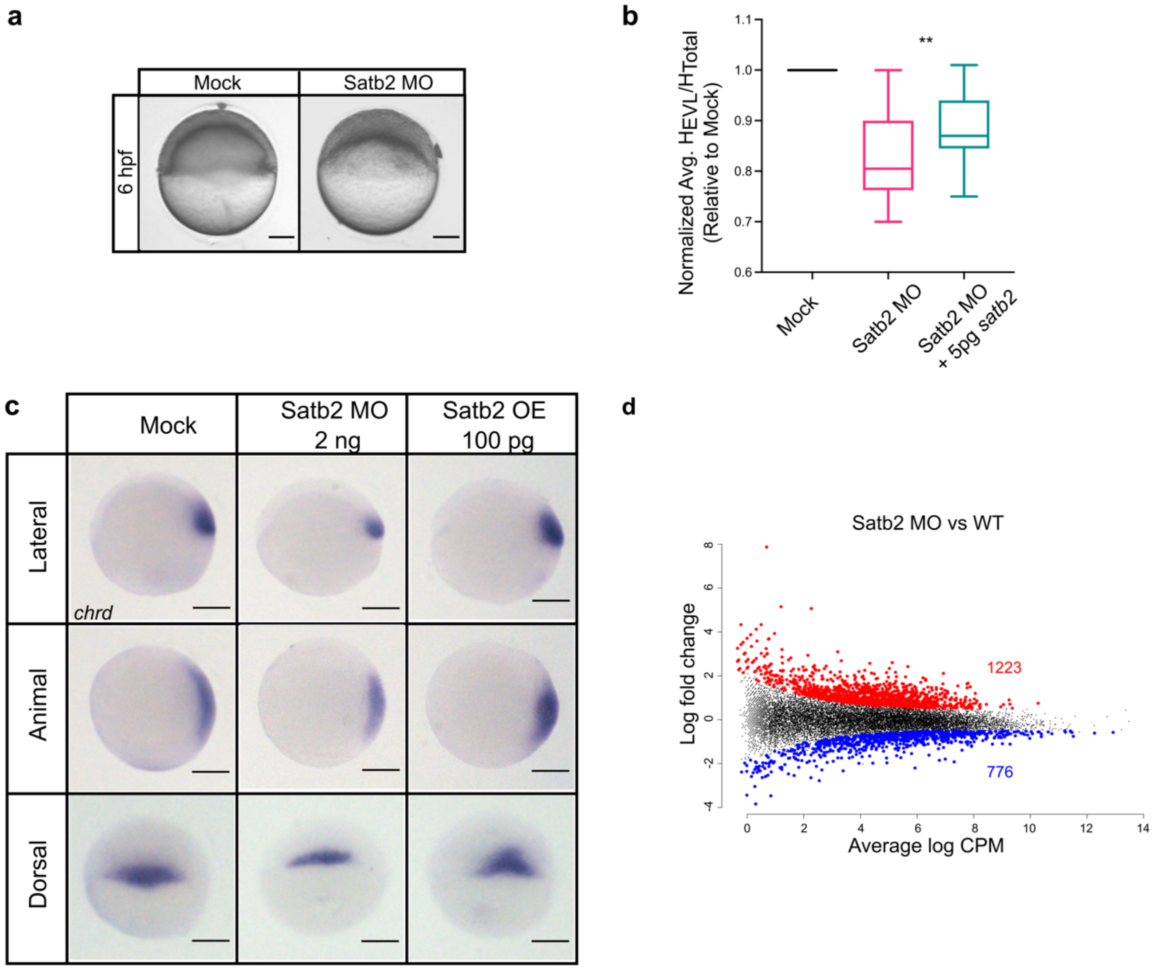

Supplementary Fig. 6. Characterization of the loss of function of maternal Satb2. a, Lateral view of zebrafish embryos at 6 hpf injected with 4 ng of control and Satb2 targeting morpholinos. N=4 biologically independent experiments. Scale bar = 100 µm. **b,** Box plot representing ratio of average height of EVL from animal pole to the total height of the embryo normalized to Mock control (n=28 embryos examined for each condition over 3 biologically independent experiments). ** signifies P value = 0.077 as determined by student's unpaired t-test. Error bar indicates +/- SEM **c,** Whole-mount *in situ* mRNA analysis for stage matched embryos showing the effect of Satb2 knockdown and over expression on the expression of dorsal marker *chrd*. n=4, N=2 biologically independent experiments. Scale bar = 100 µm. **d,** Smear plot analysis for bulk mRNA seq analysis in Control and Satb2 morpholino injected embryos at 4.5 hpf. Values in red and blue represent the number of genes differentially upregulated and downregulated respectively.

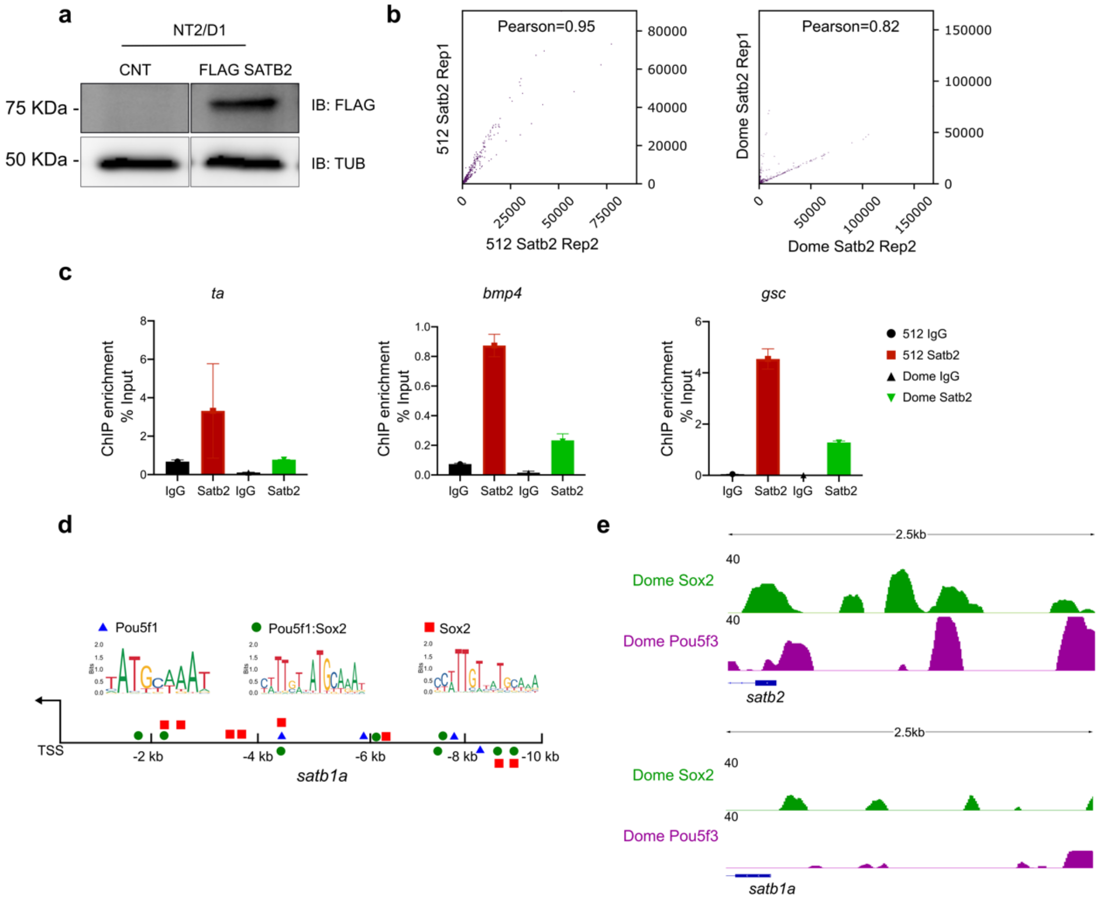

Supplementary Fig. 7. Interplay between Satb2 and pluripotency factors. a, Immunoblot analysis for validating overexpression of 3x-FLAG SATB2 in NT2/D1 cells using anti-FLAG antibody. gamma-Tubulin was used as a loading control. N=3 biologically independent experiments. **b,** Pearson correlation analysis of ChIP-sequencing datasets obtained using anti-Satb2 antibody at 512 cells stage and at the dome stage showing a high level of correlation. **c,** Quantitative relative enrichment (ChIP) represented as percent input using isotype-matched IgG and anti-Satb2 antibody for genomic locus (TSS) of negatively regulated genes *ta*, *bmp4* and *gsc*. ** indicates P-value < 0.001 as calculated by the student's two-tailed t-test, N=2 biologically independent experiments. **d,** Schematic for related protein *satb1a* promoter displaying motif sites marked for Pouf53, Pou5f3: Sox2 and Sox2. **e,** IGV snapshots of Pou5f3 occupancy on genomic loci of zebrafish *satb2* and *satb1a* respectively highlighting selective regulation by Pou5f3.

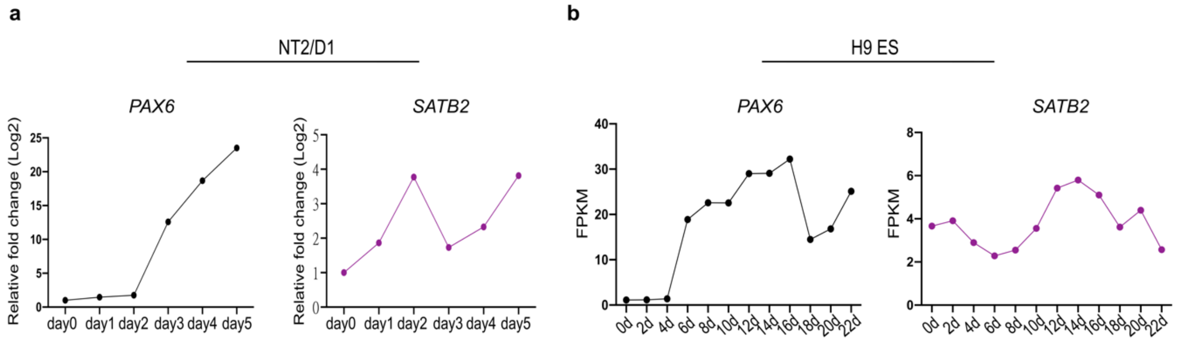

Supplementary Fig. 8. Expression pattern of human *SATB2* during differentiation to neural progenitors. a, Line graph depicting average mRNA expression levels for *PAX6* and *SATB2* upon retinoic acid induced differentiation of NT2/D1 cells (n=2). **b,** Line graph representing mRNA expression analysis of *PAX6* and *SATB2* in neural differentiation model in H9 ES cells.

Supplementary Table 1.

Key Resource table

**Reagents/Resources**	**Source**	**Identifier**
**Antibodies**		
Anti-H3K27Ac	Abcam	cat# ab4729
Anti-H3K27me3	Millipore	cat# 07-449
Anti-H3K4me3	Abcam	cat# ab8580
Anti-gamma-Tubulin	Sigma	cat# T6557
Anti-FlAG-M2	Sigma	cat# F3165
Anti-SATB2	Abcam	cat# ab34735
Anti-zebrafish Satb2	This study	cat# NA
Anti-Human Satb2	This study	cat# NA
Anti-DIG-AP Fab	Roche	cat# 1093274
Anti-Rabbit IgG	Invitrogen	cat# 31235
Anti-Rabbit-HRP conjugate	Bio Rad	cat# STAR124P
**Critical Commercial Reagents**		
Quantseq 3' mRNA seq library kit	Lexogen	cat# 015.96
SENSE mRNA-Seq Library Prep Kit	Lexogen	cat# 001.96
NEB ultra II DNA library kit	NEB	cat# E7645L
Nugen ultra II ovation DNA kit	Nugen	cat# 0344NB-A01
Single Cell 3′ Reagent Kits V3	10X Genomics	cat# PN-1000092
Hiprep PCR cleanup kit	MagBio Genomics	cat# AC-60050
SPRIselect	Beckman Coulter	cat# B23318
NextSeq 500/550 High Output Kit v2.5 (75 cycles)	Illumina	cat# 20024906
NextSeq 500/550 Mid Output Kit	Illumina	cat# 20024904

v2.5 (150 cycles)		
Dynabeads Protein A	Invitrogen	cat # 10002D
Dynabeads Protein G	Invitrogen	cat# 10004D
TDE1 Tn5	Illumina	cat# 15027865
TD Buffer	Illumina	cat# 15027866
**Experimental Models: Organisms/Strains**		
Zebrafish: Tuebingen wild-type	MPI-CBG Dresden	NA
Zebrafish: *satb2*-STOP	This study	*satb2*-E1-STOP
Zebrafish: MZ*pou5f3*	Burgess et al. 2002	*spg*
Mouse: SWR/J	The Jackson Laboratory	Stock# 000689
**Recombinant DNA clones**		
pCS2-zf.3xFLAG-*satb2*	This study	NA
pCS2-zf.*satb2*	This study	NA
pCS2-zf.mismatch-*satb2*	This study	NA
pET28b-6XHis-zf.*satb2*-STREP	This study	NA
pCMV9-3X*FLAG*-Hu.*SATB2*	This study	NA
pET28b-Cas9-His	Gagnon et al. 2014	Addgene# 47327
**Deposited Data**		
mRNA-seq	This study	NCBI BioProject ID PRJNA657343
Single cell RNAseq	This study	NCBI BioProject ID PRJNA657343
ChIP-seq	This study	NCBI BioProject ID PRJNA657343
ATAC-seq	This study	NCBI BioProject ID PRJNA657343
**Software and Algorithms**		

Trimmomatic 0.39	Bolger et al. 2014	https://github.com/timflutre/trimmomatic
BWA	Li H. and Durbin R. (2009)	http://bio-bwa.sourceforge.net/
STAR2.4.2	Dobin et al. 2013	https://github.com/alexdobin/STAR
BBMap	Bushnell B. 2014	https://sourceforge.net/projects/bbmap/
Deeptools	Ramirez et al. 2016	https://github.com/deeptools/deepTools
HOMER (v.4.4)	Heinz et al. 2010	http://homer.ucsd.edu/homer/index.html
MACS2 (v2.1.0)	Zhang et al. 2008	https://github.com/taoliu/MACS
FeatureCounts (v1.4.6-p4)	Liao et al. 2014	http://bioinf.wehi.edu.au/featureCounts
edgeR	Robinson et al. 2010	https://bioconductor.org/packages/release/bioc/html/edgeR.html
Diffbind	Stark and Brown 2011	https://bioconductor.org/packages/release/bioc/html/DiffBind.html
Seurat 3.0	Butler et al. 2018	https://satijalab.org/seurat/
NucleoATAC	Schep et al. 2015	https://github.com/GreenleafLab/NucleoATAC
Fiji	Schindelin et al. 2012	https://fiji.sc/
GraphPad Prism	GraphPad software	https://www.graphpad.com/scientific-software/prism/
R version 3.6.1	The R Project for Statistical Computing	https://www.r-project.org/
BioRender	BiorRender	https://biorender.com/

Supplementary Table 2.

Oligo Name	Oligo sequence	Purpose
sgRNA oligo sequence targeting *satb2* (bold letters)	ATTTAGGTGACACTATA**GGGCCCCGTTGTGACGACTG**TTTTAGAGCTAGAAATAGCAAG	CRISPR-Cas9 (Related to Fig. 1b)
Constant oligo for synthesis of sgDNA	AAAAGCACCGACTCGGTGCCACTTTTTCAAGTTGATAACGGACTAGCCTTATTTTAACTTGCTATTTCTAGCTCTAAAAC	CRISPR-Cas9 (Related to Fig. 1b)
STOP cassette with homology arms (lower case)	cagccccactggtcaccgcagGTCATGGCGTTTAAACCTTAATTAAGCTGTTGTAGtcgtcacaacggggccccacwere	CRISPR-Cas9 (Related to Fig. 1b)
z. Satb2 mutant screen FWD	GGAGGAGAGAGTCCTCGACTG	Genotyping (Related to Fig. 1c)
z. Satb2 mutant screen REV	GTTGCAGCATGTTTCAGATGAT3	Genotyping (Related to Fig. 1c)
z. *id2a* FWD	AGGCGAGTCTTTTCAACGAA	WISH (Related to Fig. 2e)
z. *id2a* REV	agtaatacgactcactataggGATATTTGACGGGACGCTGAG	WISH (Related to Fig. 2e)
z. *sox9a* FWD	CGCAGAATCTCCTCGACCC	WISH (Related to Fig. 2e)
z. *sox9a* REV	gagtaatacgactcactataggGGGGACTGGCCTGAGTGTTCG	WISH (Related to Fig. 2e)
z. *zic1* FWD	TTCTTTTTCGCAATCGGGGC	WISH (Related to Fig. 2e)
z. *zic1* REV	gagtaatacgactcactataggGAAGGTTTTCACCTGTGTGTGT	WISH (Related to Fig. 2e)
z. *mych* FWD	CTCCGACATAGACACGCAGA	WISH (Related to Supp Fig. 5d)
z. *mych* REV	gagtaatacgactcactatagggGTCTGGCTTTCAGCTGTTCC	WISH (Related to Supp Fig. 5d)
z. *vox* FWD	AGACGGAGAGCAGCAAAGAG	WISH (Related to

		Supp Fig. 5d)
z. *vox* REV	gagtaatacgactcactatagggGAGGATGAGGATGGTGAGGA	WISH (Related to Supp Fig. 5d)
z. *foxd5* FWD	TCTCCAACCATGACCCTCTC	WISH (Related to Supp Fig. 5d)
z. *foxd5* REV	gagtaatacgactcactatagggACCTCTGGGTTTTGTGTTCG	WISH (Related to Supp Fig. 5d)
z. *mxtx2* FWD	TCTGCAAGAGAGCTGCAAAA	WISH (Related to Supp Fig. 5d)
z. *mxtx2* REV	gagtaatacgactcactatagggAGGCACAGATGGAGAGCAGT	WISH (Related to Supp Fig. 5d)
z. *chrd* FWD	GTGGCCGCTTTTACTCTG	WISH (Related to Supp Fig. 6c)
z. *chrd* REV	GTGAGGTTTCGGCACATTCT	WISH (Related to Supp Fig. 6c)
z. *pou5f1* FWD	CGGAAAGATGACGGAGAGAG	WISH (Related to Fig. 6b)
z. *pou5f1* REV	gagtaatacgactcactatagggAGCTCTTTCGCAAACTGCTC	WISH (Related to Fig. 6b)
Danio_*satb2* FWD (5’→3’)	TCTTTTTGCAGGATCATGGAGCGTGGTGGAGGAG	Cloning (Related to Fig. 5b)
Danio_*satb2* REV (5’→3’)	CATGTCTGGATCTACTCTCTGATAGTCTGCTTGATCTCTG	Cloning (Related to Fig. 5b)
3xFLAG-Danio_*satb2* FWD (5’→3’)	TTGTTCTTTTTGCAGGACTACAAAGACCATGGTGATTATAAAGATCATGACATCGATTACAAGGATGACGATGACAAGATGGAGCGTGGTGGAGG	Cloning (Related to Fig. 5b)
3xFLAG-Danio_*satb2* REV (5’→3’)	CGAATCGATGGGATCTTATCTCTGATAGTCTGCTTGATCT	Cloning (Related to Fig. 5b)

Mismatch-Danio_*satb2* FWD (5'→3') (mismatches are highlighted by red text)	TTGTTCTTTTGCAGATGGA**A**CGTGG**C**GG**C**GGAGA**ATCA**CCTCGA	Cloning (Related to Supp Fig. 6b)
6xHis-Danio_*satb2*-strep FWD (5'→3')	GAACTGAGGATGAGACCATAGTCTCTGATAGTCTGCT	Cloning (Related to Supp Fig. 3a)
6xHis-Danio_*satb2*-strep REV (5'→3')	GTATTTCCAAGGCTCGATGGAACGGGGTGGAGGAGA	Cloning (Related to Supp Fig. 3a)
3xFLAG-Human_*SATB2* FWD (5'→3')	GCGAATTCGATGGAGCGGCGGAGCGAGAGCCCG	Cloning (Related to Fig. 6e)
3xFLAG-Human_*SATB2* REV (5'→3')	GCGTCTAGATTATCTCTGGTCAATTTCGGCAGG	Cloning (Related to Fig. 6e)
satb2 MO1 (targeting translation initiation) (5'→3')	GACTCTCTCCTCCACCACGCTCCAT	Antisense knockdown Cloning (Related to Fig. 5d)
satb2 MO2 (targeting splicing) (5'→3')	TGTGAAGTGCCTGATGAGAAAAGAA	Antisense knockdown Cloning (Related to Fig. 5d)
satb2 mismatch control MO (comtrol MO) (5'→3')	GA**G**T**G**TCTC**G**TCCAC**G**AC**C**CTCCAT	Antisense knockdown Cloning (Related to Fig. 5d)
z. *pou5f3* FWD	AGGGCAGGAATGACAAAATG	ChIP-qPCR (Related to Fig. 6g)
z. *pou5f3* REV	CACCCGAGACAATAAGCTACAG	ChIP-qPCR (Related to Fig. 6g)
z. *sox19b* FWD	ACCCGATGGACAAAGTCAAG	ChIP-qPCR (Related to Fig. 6g)
z. *sox19b* REV	AGGGCAGGAATGACAAAATG	ChIP-qPCR (Related to Fig. 6g)
z. *ta* FWD	GCGCTGTCAAAGCAACAGTA	ChIP-qPCR

		(Related to Supp Fig. 7c)
z. *ta* REV	GCGTCTTCAAGCGAAAGTTTA	ChIP-qPCR (Related to Supp Fig. 7c)
z. *gsc* FWD	TCGCGGTTTTTGTCACTATG	ChIP-qPCR (Related to Supp Fig. 7c)
z. *gsc* REV	GGATTCCGTCAAGTTGGAGA	ChIP-qPCR (Related to Supp Fig. 7c)
z. *bmp4* FWD	TTTATTTCAGAACAGGATTACGC	ChIP-qPCR (Related to Supp Fig. 7c)
z. *bmp4* REV	GACGTCTTCTCCGTTTGACC	ChIP-qPCR (Related to Supp Fig. 7c)
z. *satb2* FWD	CAAGAGTTTGGTCGCTGGTA	qRT-PCR (Related to Fig. 6j)
z. *satb2* REV	CGCTGGGCTAATACACAGAA	qRT-PCR (Related to Fig. 6j)
z. *ef1a* FWD	CTTCTCAGGCTGACTGTGC	qRT-PCR (Related to Fig. 6j)
z. *ef1a* REV	ACGATCAGCTGTTTCACTCC	qRT-PCR (Related to Fig. 6j)
Hu. *OCT4* FWD	AGCAAAACCCGGAGGAGT	qRT-PCR (Related to Fig. 6e)
Hu. *OCT4* REV	CCACATCGGCCTGTGTATATC	qRT-PCR (Related to Fig. 6e)
Hu. *GAPDH* FWD	CTGCACCACCAACTGCTTAG	qRT-PCR (Related to Fig. 6e)
Hu. *GAPDH* REV	GTCTTCTGGGTGGCAGTGAT	qRT-PCR (Related to Fig. 6e)
