# Supplementary material for: Satb2 acts as a gatekeeper for major developmental transitions during early vertebrate embryogenesis
Source: Nat Commun. 2021 Oct 19;12:6094. doi: 10.1038/s41467-021-26234-7 (PMC8526749; doi:10.1038/s41467-021-26234-7)
Supplement: Supplementary file 3 — Reporting Summary [file 41467_2021_26234_MOESM3_ESM.pdf]

nature portfolio

Corresponding author(s): Prof. Sanjeev Galande

Last updated by author(s): Aug 31, 2021

Reporting Summary

Nature Portfolio wishes to improve the reproducibility of the work that we publish. This form provides structure for consistency and transparency in reporting. For further information on Nature Portfolio policies, see our Editorial Policies and the Editorial Policy Checklist.

Statistics

For all statistical analyses, confirm that the following items are present in the figure legend, table legend, main text, or Methods section.

n/a	Confirmed	
☐	☒	The exact sample size (*n*) for each experimental group/condition, given as a discrete number and unit of measurement
☐	☒	A statement on whether measurements were taken from distinct samples or whether the same sample was measured repeatedly
☐	☒	The statistical test(s) used AND whether they are one- or two-sided<br>*Only common tests should be described solely by name; describe more complex techniques in the Methods section.*
☒	☐	A description of all covariates tested
☒	☐	A description of any assumptions or corrections, such as tests of normality and adjustment for multiple comparisons
☐	☒	A full description of the statistical parameters including central tendency (e.g. means) or other basic estimates (e.g. regression coefficient) AND variation (e.g. standard deviation) or associated estimates of uncertainty (e.g. confidence intervals)
☐	☒	For null hypothesis testing, the test statistic (e.g. *F*, *t*, *r*) with confidence intervals, effect sizes, degrees of freedom and *P* value noted<br>*Give P values as exact values whenever suitable.*
☒	☐	For Bayesian analysis, information on the choice of priors and Markov chain Monte Carlo settings
☒	☐	For hierarchical and complex designs, identification of the appropriate level for tests and full reporting of outcomes
☐	☒	Estimates of effect sizes (e.g. Cohen's *d*, Pearson's *r*), indicating how they were calculated

Our web collection on statistics for biologists contains articles on many of the points above.

Software and code

Policy information about availability of computer code

Data collection	Data collection were performed using imaging softwares Leica suite and Illumina sequencing manager.
Data analysis	Image data analysis was performed ImageJ. Sequencing data were demultiplexed using bcl2fastq tool. Sequencing reads were aligned using HISAT2, BWA, Bowtie2 and STAR aligner as indicated in Methods section. Downstream Analysis was performed using MACS2, HOMER and Deeptools.

For manuscripts utilizing custom algorithms or software that are central to the research but not yet described in published literature, software must be made available to editors and reviewers. We strongly encourage code deposition in a community repository (e.g. GitHub). See the Nature Portfolio guidelines for submitting code & software for further information.

Data

Policy information about availability of data

All manuscripts must include a data availability statement. This statement should provide the following information, where applicable:

- Accession codes, unique identifiers, or web links for publicly available datasets
- A description of any restrictions on data availability
- For clinical datasets or third party data, please ensure that the statement adheres to our policy

All the raw data generated in this study, including RNA-seq, ATAC-seq, ChIP-seq and scRNAseq data can be accessed on GEO under accession ID GSE183002. All other relevant source data supporting the key findings of this study are provided with this paper.
All the custom computational codes used in this study are available on Zenodo with DOI: 10.5281/zenodo.5336938 and upon request from the first (saurabh.j.pradhan@gmail.com) or the corresponding author (sanjeev@iiserpune.ac.in).

Field-specific reporting

Please select the one below that is the best fit for your research. If you are not sure, read the appropriate sections before making your selection.

- [x] Life sciences
- [ ] Behavioural & social sciences
- [ ] Ecological, evolutionary & environmental sciences

For a reference copy of the document with all sections, see nature.com/documents/nr-reporting-summary-flat.pdf

Life sciences study design

All studies must disclose on these points even when the disclosure is negative.

Sample size	Sample size for each experiment, number of embryos, number of cells were determined as per maximum availability of the source material.
Data exclusions	Sequencing reads or cells containing poorly aligned reads were discarded prior to analysis. Quality of sequencing reads and single cells were determined using Trimmomatic and CellRanger pipelines.
Replication	All the experiments were performed in replicates and number of replicates are clearly mentioned in respective figure legends. No replicate were removed intentionally. All replicates showed strong correlation.
Randomization	Randomization of the samples is not applicable for this study.
Blinding	Blinding is not applicable for this study.

Reporting for specific materials, systems and methods

We require information from authors about some types of materials, experimental systems and methods used in many studies. Here, indicate whether each material, system or method listed is relevant to your study. If you are not sure if a list item applies to your research, read the appropriate section before selecting a response.

Materials & experimental systems

n/a	Involved in the study	
	x	Antibodies
	x	Eukaryotic cell lines
x		Palaeontology and archaeology
	x	Animals and other organisms
x		Human research participants
x		Clinical data
x		Dual use research of concern

Methods

n/a	Involved in the study	
	x	ChIP-seq
	x	Flow cytometry
x		MRI-based neuroimaging

Antibodies

Antibodies used	Anti-H3K27Ac Abcam cat# ab4729, Anti-H3K27me3 Millipore cat# 07-449, Anti-H3K4me3 Abcam cat# ab8580, Anti-gamma-Tubulin Sigma cat# T6557, Anti-FlAG-M2 Sigma cat# F3165, Anti-SATB2 Abcam cat# ab34735, Anti-zebrafish Satb2 This study cat# NA, Anti-Human Satb2 This study cat# NA, Anti-DIG-AP Fab Roche cat# 1093274, Anti-Rabbit IgG Invitrogen cat# 31235, Anti-Rabbit-HRP conjugate Bio Rad cat# STAR124P
Validation	All the commercial antibodies are pre-validated and used in previous publications. In house generated antibodies against zeebrafish and human Satb2 were validated using Immunoblotting, Immunoprecipitation and knockouts.

Eukaryotic cell lines

Policy information about cell lines

Cell line source(s)	NT2/D1 (NTERA2-clone D1) were a kind gift from Dr. Peter Andrews, University of Sheffield, UK.
Authentication	Cell line authentication was performed using karyotyping
Mycoplasma contamination	Cell lines were routinely tested for mycoplasma and other contaminants.
Commonly misidentified lines (See ICLAC register)	Not applicable

Animals and other organisms

Policy information about studies involving animals; ARRIVE guidelines recommended for reporting animal research

Laboratory animals	Zebrafish: Tuebingen wild-type MPI-CBG Dresden, Zebrafish: satb2-STOP This study , IISER-Pune, Zebrafish: MZpou5f3 Burgess et al. 2002, Mouse: SWR/J The Jackson Laboratory Stock# 000689
Wild animals	No wild animals were used in this study.
Field-collected samples	No field collected samples were used in this study
Ethics oversight	All the experimental procedures involving zebrafish were carried out in accordance with the guidelines from the institutional animal ethics committee at IISER Pune and IST Austria. All the mouse experiments were carried out at TIFR, Mumbai adhering to Institutional ethics guidelines.

Note that full information on the approval of the study protocol must also be provided in the manuscript.

ChIP-seq

Data deposition

- [x] Confirm that both raw and final processed data have been deposited in a public database such as GEO.
- [ ] Confirm that you have deposited or provided access to graph files (e.g. BED files) for the called peaks.

Data access links
May remain private before publication.

Dataset is publicly accessible on GEO under accession id GSE183002
https://www.ncbi.nlm.nih.gov/geo/query/acc.cgi?acc=GSE183002

Files in database submission

Raw and processed data for ChIP-seq
Raw and processed data RNA-seq fastq files,
Raw and processed data ATAC-seq fastq files,
Raw and processed data single cell fastq and barcode files.

GSM5548460 Quantseq_80epi_WT_BR1
GSM5548461 Quantseq_80epi_WT_BR2
GSM5548462 Quantseq_80epi_MUT_BR1
GSM5548463 Quantseq_80epi_MUT_Rep2
GSM5548464 Quantseq_6som_WT_BR1
GSM5548465 Quantseq_6som_WT_BR2
GSM5548466 Quantseq_6som_SATB2_MUT_BR1
GSM5548467 Quantseq_6som_SATB2_MUT_BR2
GSM5548468 Quantseq_14som_WT_BR1
GSM5548469 Quantseq_14som_WT_BR2
GSM5548470 Quantseq_14som_SATB2_MUT_BR1
GSM5548471 Quantseq_14som_SATB2_MUT_BR2
GSM5548472 ChIPseq_512_Input
GSM5548473 ChIPseq_512-SATB2-BR1
GSM5548474 ChIPseq_512-SATB2-BR2
GSM5548475 ChIPseq_512-SATB2-BR3
GSM5548476 ChIPseq_Dome_Input
GSM5548477 ChIPseq_Dome-SATB2-BR1
GSM5548478 ChIPseq_Dome-SATB2-BR2
GSM5548479 ChIPseq_Dome-SATB2-BR3
GSM5548480 ChIPseq_80epi_Input
GSM5548481 ChIPseq_80epi-SATB2-BR1
GSM5548482 ChIPseq_80epi-SATB2-BR2
GSM5548483 ChIPseq_6som_Input
GSM5548484 ChIPseq_6som-SATB2-BR1
GSM5548485 ChIPseq_6som-SATB2-BR2
GSM5548486 ChIPseq_14som_Input
GSM5548487 ChIPseq_14som-SATB2-BR1
GSM5548488 ChIPseq_14som-SATB2-BR2
GSM5548489 ChIPseq_14som-SATB2-BR3
GSM5548490 ChIPseq_Dome-SATB2OE_SATB2
GSM5548491 ChIPseq_Dome-SATB2OE_FLAG
GSM5548492 ChIPseq_Dome-CNT_H3K27Ac
GSM5548493 ChIPseq_Dome-CNT_H3K27me3
GSM5548494 ChIPseq_Dome-CNT_H3K4me3
GSM5548495 ChIPseq_Dome-SATB2OE_H3K27Ac

GSM5548496 ChIPseq_Dome-SATB2OE_H3K27me3
GSM5548497 ChIPseq_Dome-SATB2OE_H3K4me3
GSM5548498 ChIPseq_E9_5_HeadInput
GSM5548499 ChIPseq_E9_5_HeadSATB2_BR1
GSM5548500 ChIPseq_E9_5_HeadSATB2_BR2
GSM5548501 ChIPseq_E9_5_TrunkInput
GSM5548502 ChIPseq_E9_5_TrunkSATB2_BR1
GSM5548503 ChIPseq_E9_5_TrunkSATB2_BR2
GSM5548504 ChIPseq_E13_5_dTelInput
GSM5548505 ChIPseq_E13_5_dTelSATB2_BR1
GSM5548506 ChIPseq_E13_5_dTelSATB2_BR2
GSM5548507 ATACseq_WT_Dome_BR1
GSM5548508 ATACseq_WT_Dome_BR2
GSM5548509 ATACseq_SATB2OE_Dome_BR1
GSM5548510 ATACseq_SATB2OE_Dome_BR2
GSM5548511 ATACseq_WT_Som_14_BR1
GSM5548512 ATACseq_WT_Som_14_BR2
GSM5548513 ATACseq_SATB2mut_Som_14_BR1
GSM5548514 ATACseq_SATB2mut_Som_14_BR2
GSM5548515 RNAseq_Dome_Ctrl_BR1
GSM5548516 RNAseq_Dome_Ctrl_BR2
GSM5548517 RNAseq_Dome_Ctrl_BR3
GSM5548518 RNAseq_Dome_SATB2OE_BR1
GSM5548519 RNAseq_Dome_SATB2OE_BR2
GSM5548520 RNAseq_Dome_SATB2OE_BR3
GSM5548521 RNAseq_Dome_Satb2_Momix_BR1
GSM5548522 RNAseq_Dome_Satb2_Momix_BR2
GSM5548523 RNAseq_Dome_Satb2_Momix_BR3
GSM5548524 scRNAseq_FLAG_SATB2
GSM5548525 Sox10_Negative_input
GSM5548526 Satb2_ChIPseq_Sox10_neg_Rep1
GSM5548527 Satb2_ChIPseq_Sox10_neg_Rep2
GSM5548528 Satb2_ChIPseq_Sox10_neg_Rep3
GSM5548529 Sox10_Positive_input
GSM5548530 Satb2_ChIPseq_Sox10_pos_Rep1
GSM5548531 Satb2_ChIPseq_Sox10_pos_Rep2
GSM5548532 Satb2_ChIPseq_Sox10_pos_Rep3
GSM5548533 WT_sibs_14ss_scRNAseq
GSM5548534 Satb2_MUT_14ss_scRNAseq

Genome browser session
(e.g. UCSC)

Provide a link to an anonymized genome browser session for "Initial submission" and "Revised version" documents only, to enable peer review. Write "no longer applicable" for "Final submission" documents.

Methodology

Replicates

Experiments were performed in minimum two biological experiments. Exact number of replicates are mentioned in respective figure legends.

Sequencing depth

High Sequencing depth was obtained for each ChIPseq datasets. Minimum 25-30 million mappable reads were obtained.

Antibodies

Anti-H3K27Ac Abcam cat# ab4729
Anti-H3K27me3 Millipore cat# 07-449
Anti-H3K4me3 Abcam cat# ab8580
Anti-gamma-Tubulin Sigma cat# T6557
Anti-FlAG-M2 Sigma cat# F3165
Anti-SATB2 Abcam cat# ab34735
Anti-zebrafish Satb2 This study cat# NA
Anti-Human Satb2 This study cat# NA

Peak calling parameters

For ChIP-seq default parameters of MACS2 and HOMER were used. For ATAC-seq experiments following parameters were used. Peak calling was performed using macs2 callpeak -f BEDPE -q 0.05 --nomodel --extsize 200 --gsize 1.3e9 --keep-dup 2

Data quality	Data quality were determined using fastQC, Trimmomatic and Deeptools. Reproducibility between datasets were determined using Pearson correlation from Deeptools.
Software	Sequencing reads were trimmed using TrimmomaticPE for Truseq2:PE adapters and reads with quality greater than phred 33 were retained. Quality of sequencing reads were determined using fastQC. High quality sequencing reads were aligned to zebrafish danRer10 genome version using default parameters of BWA88. Aligned reads were subsampled to 40 million reads in each sample using Bbmap. Correlation between each replicate was estimated using multiBamSummary and replicates showing very high Pearson correlation (> 0.7) were used for further analysis. Peak calling was performed using macs2 with default parameters and q value 0.05. Consensus set of peaks from biological replicates were extracted using a custom R script from Roman Cheplyaka (https://ro-che.info/articles/2018-07-11-chip-seq-consensus). Bigwig files were generated first using bamCoverage normalizing to RPKM and then subtracting Input signals using bamCompare utilities from deepTools 3.3.2 and used for visualization with Integrative Genomics Viewer (IGV).

Flow Cytometry

Plots

Confirm that:

- [x] The axis labels state the marker and fluorochrome used (e.g. CD4-FITC).
- [x] The axis scales are clearly visible. Include numbers along axes only for bottom left plot of group (a 'group' is an analysis of identical markers).
- [x] All plots are contour plots with outliers or pseudocolor plots.
- [x] A numerical value for number of cells or percentage (with statistics) is provided.

Methodology

Sample preparation	To perform neural crest specific ChIP-seq for Satb2, Sox10pos and Sox10neg cells were isolated from Tg: sox10:GFP transgenic fish using high speed flow cytometry sorting . Cells were directly lysed in the lysis buffer to isolate chromatin by sonication. ChIP experiment was performed as described in the above section.
Instrument	FACS ARIA III, (Becton Dickinson)
Software	FlowJo
Cell population abundance	Sox10 positive neural crest cells comprises about 5-7% of total population at 14 somites stage. Exact percentage is provided in supplementary figure.
Gating strategy	live single cell population were defined as parent population. Next Sox10:egfp negative and positive cells were defined as subpopulation and gating strategy is provided in supplementary figure.

- [x] Tick this box to confirm that a figure exemplifying the gating strategy is provided in the Supplementary Information.
