# Supplementary material for: Satb2 acts as a gatekeeper for major developmental transitions during early vertebrate embryogenesis
Source: Nat Commun. 2021 Oct 19;12:6094. doi: 10.1038/s41467-021-26234-7 (PMC8526749; doi:10.1038/s41467-021-26234-7)
Supplement: Supplementary file 4 — Source Data [file 41467_2021_26234_MOESM4_ESM.zip › Pradhan_etal_SourceData/Pradhan_et.al.Source_Data2.pptx]

## Slide 1
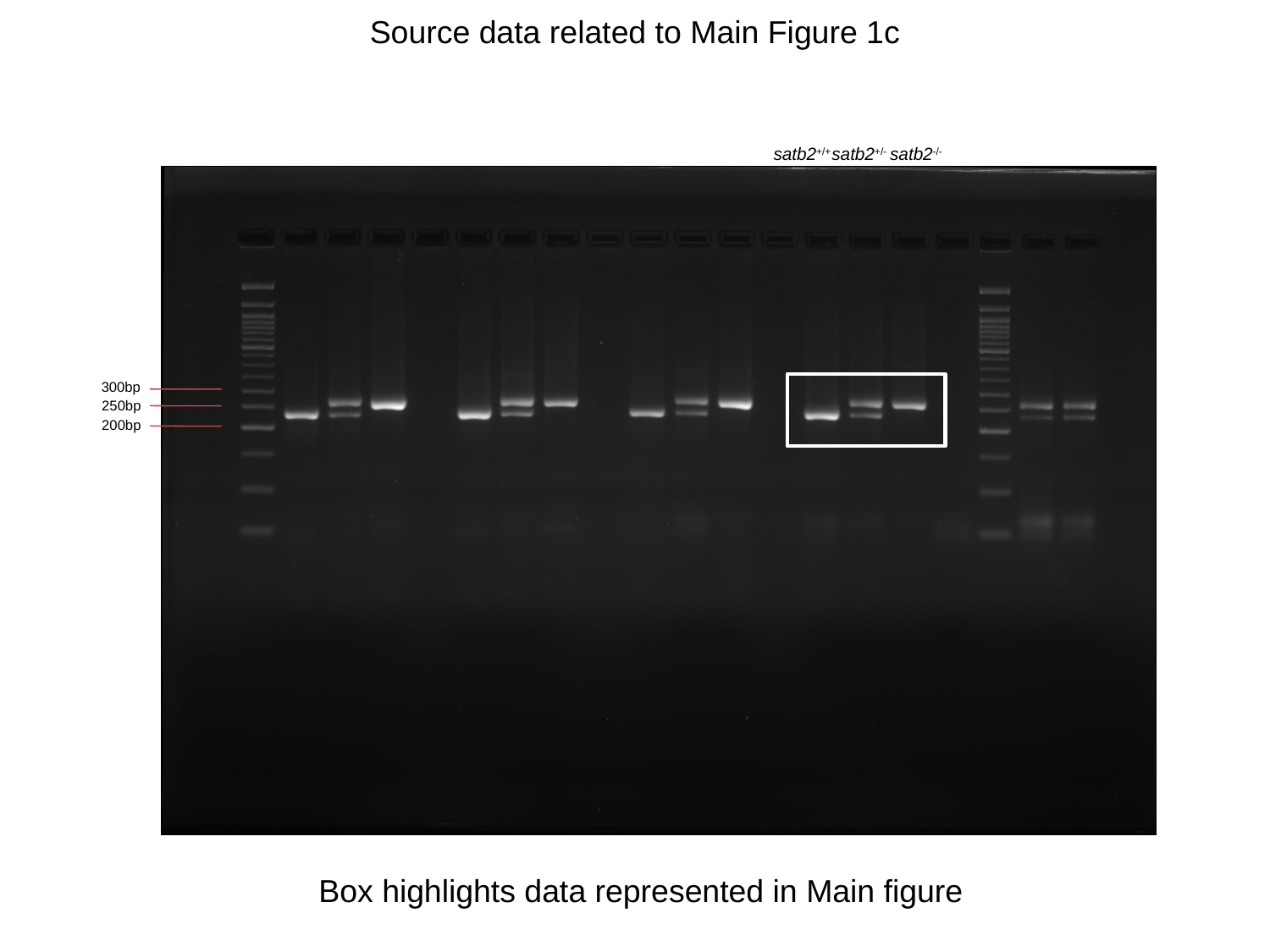

Source data related to Main Figure 1c
satb2+/+
satb2+/-
satb2-/-
300bp
250bp
200bp
Box highlights data represented in Main figure

## Slide 2
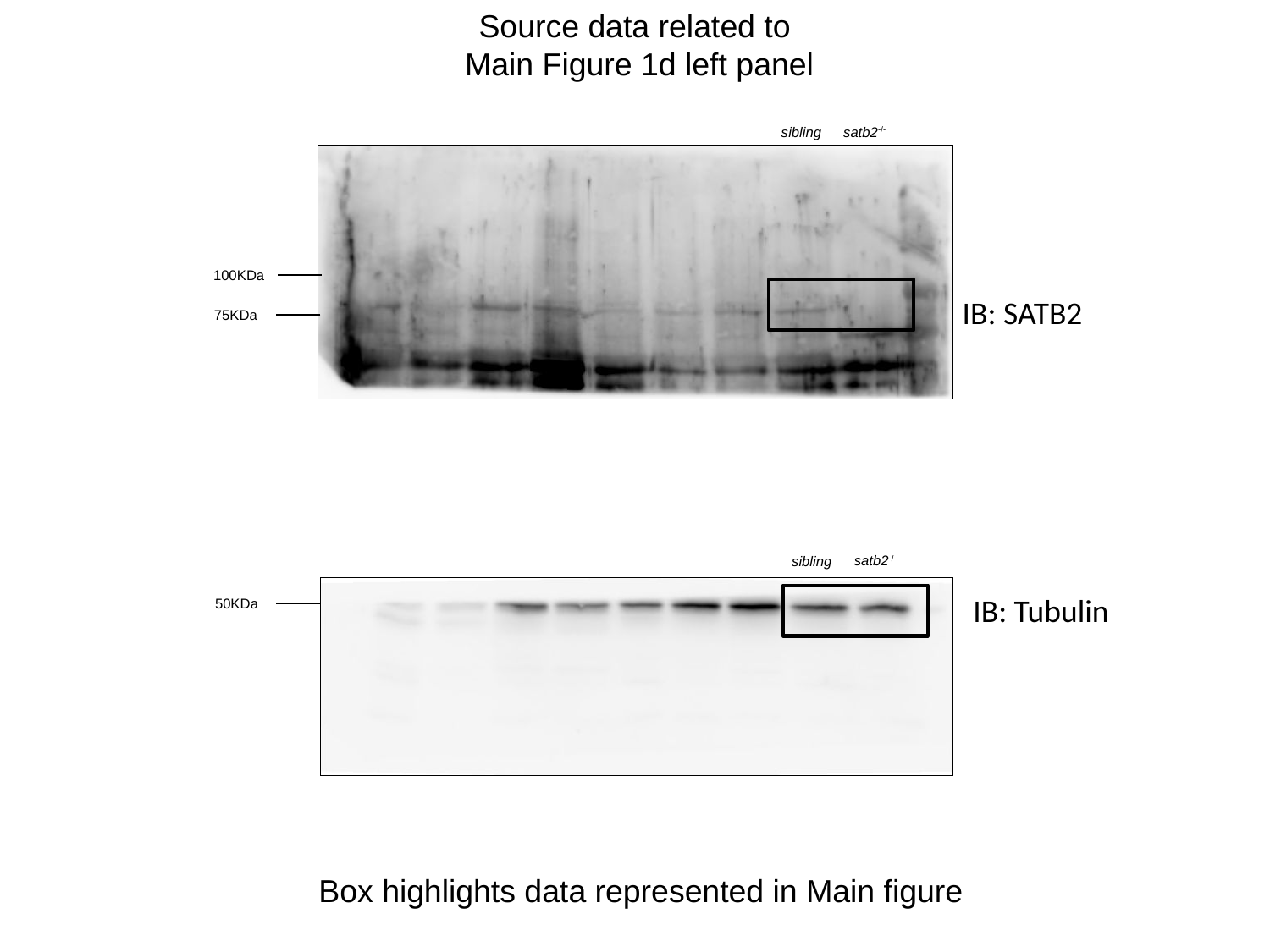

Source data related to
Main Figure 1d left panel
sibling
satb2-/-
100KDa
IB: SATB2
75KDa
satb2-/-
sibling
IB: Tubulin
50KDa
Box highlights data represented in Main figure

## Slide 3
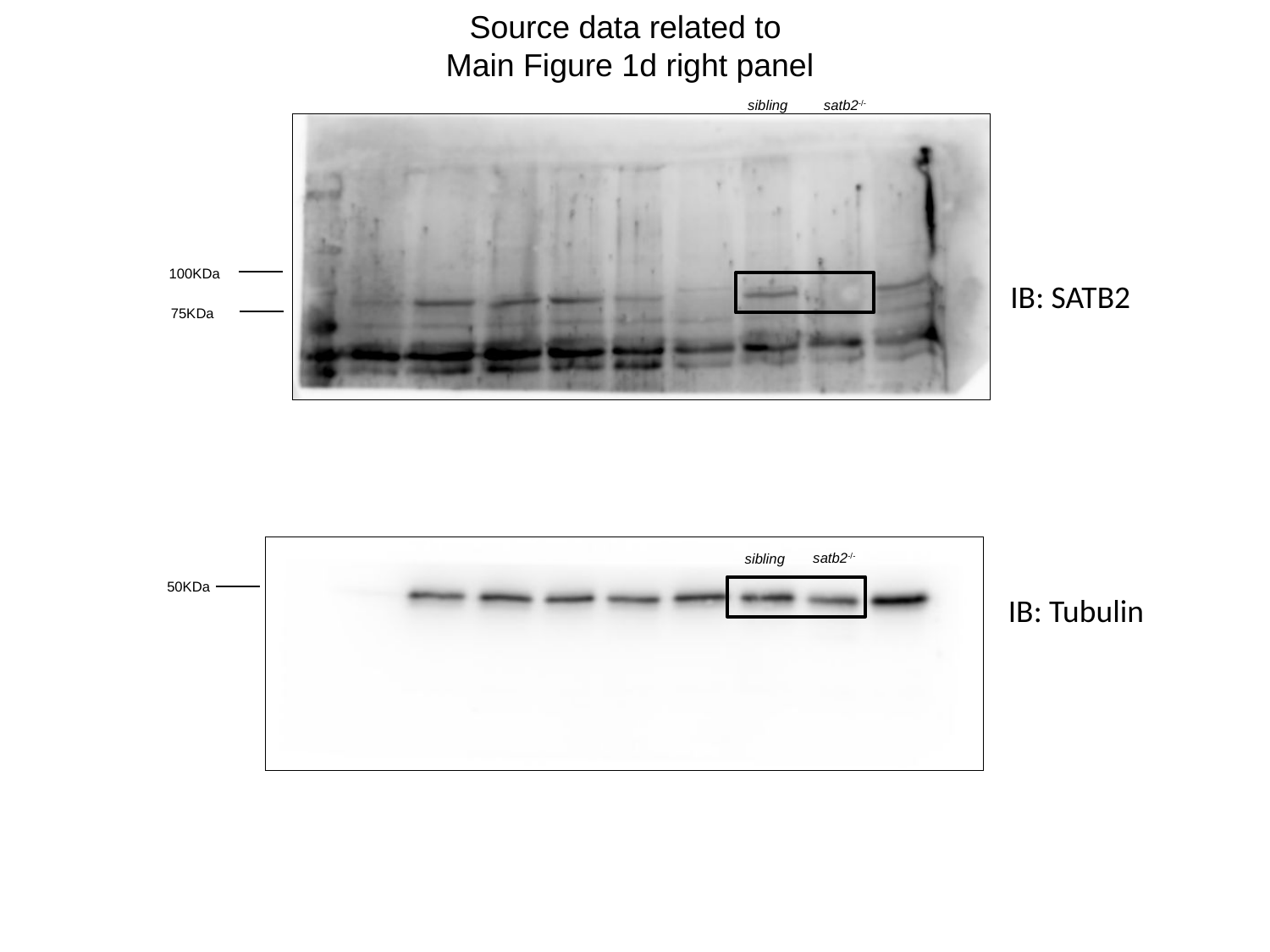

Source data related to
Main Figure 1d right panel
sibling
satb2-/-
100KDa
IB: SATB2
75KDa
satb2-/-
sibling
50KDa
IB: Tubulin

## Slide 4
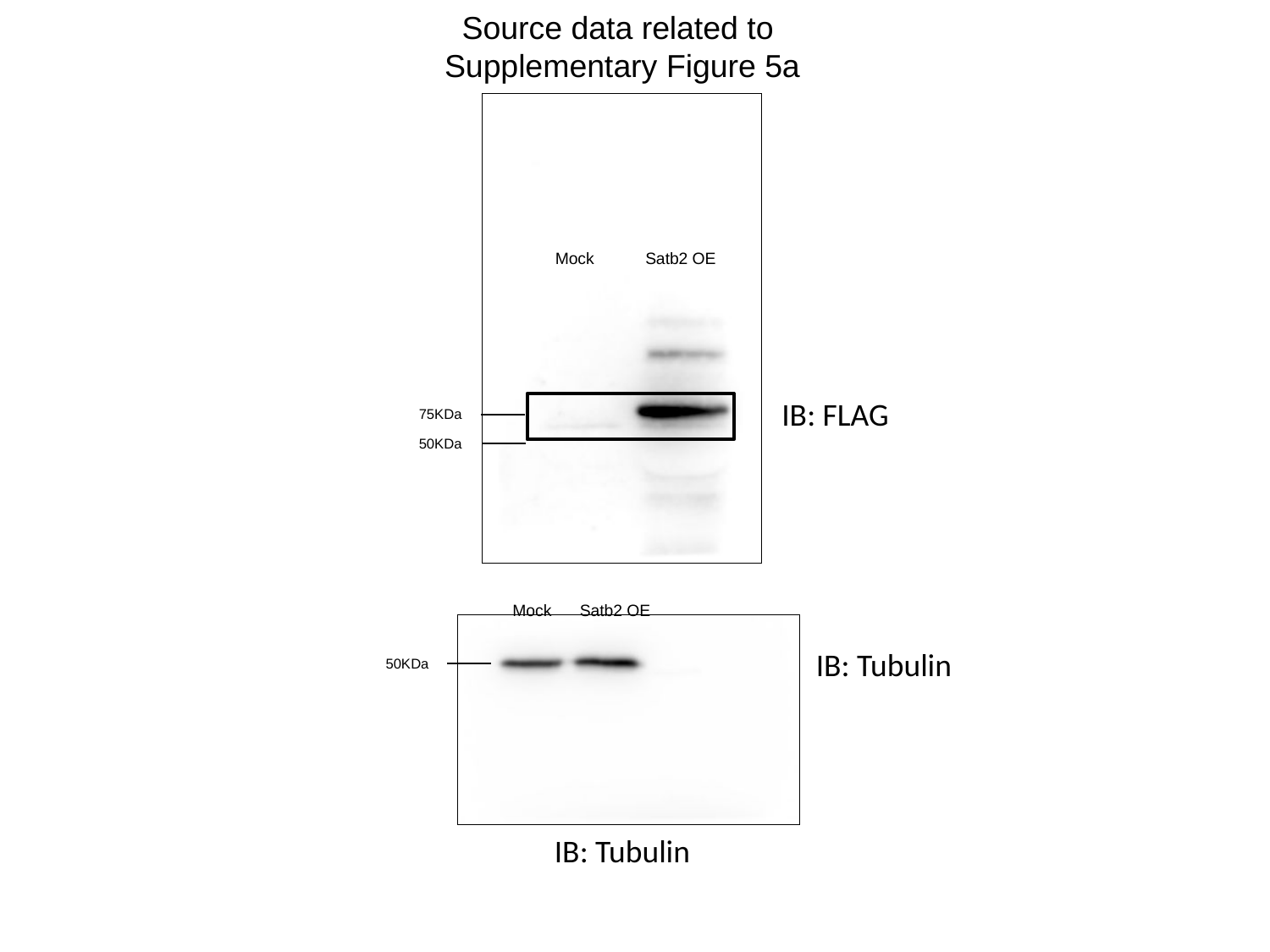

Source data related to
Supplementary Figure 5a
Mock
Satb2 OE
IB: FLAG
75KDa
50KDa
Mock
Satb2 OE
IB: Tubulin
50KDa
IB: Tubulin

## Slide 5
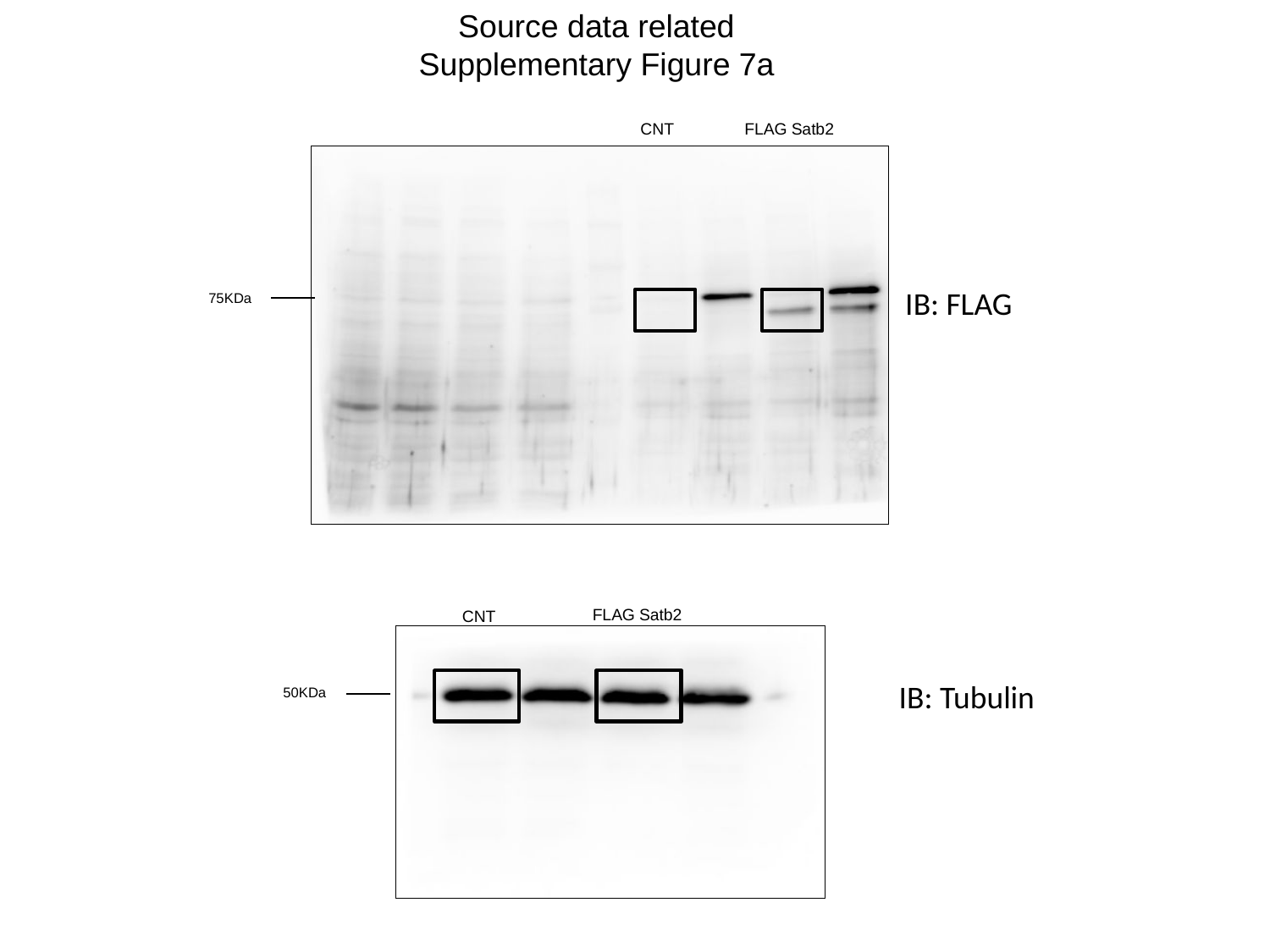

Source data related Supplementary Figure 7a
CNT
FLAG Satb2
IB: FLAG
75KDa
FLAG Satb2
CNT
IB: Tubulin
50KDa
